# Clinical Challenges in Acute Cholecystitis: Endoscopic Drainage Strategies (EUS-GBD vs. ET-GBD) in Patients with Surgical Contraindications

**DOI:** 10.3390/jcm15145536

**Published:** 2026-07-15

**Authors:** Dong Wook Lee, Chang Min Cho

**Affiliations:** Department of Internal Medicine, School of Medicine, Kyungpook National University, Daegu 41944, Republic of Korea; storm5333@naver.com

**Keywords:** acute cholecystitis, endoscopic transpapillary gallbladder drainage, endoscopic ultrasound-guided gallbladder drainage, lumen-apposing metal stent, surgical high-risk patients

## Abstract

Acute cholecystitis (AC) is one of the most prevalent gastrointestinal emergencies, with laparoscopic cholecystectomy representing the definitive treatment per current guidelines. However, a substantial proportion of patients—particularly elderly individuals with major comorbidities, multiorgan dysfunction, or advanced malignancy—are considered poor surgical candidates in whom operative intervention carries prohibitive risk. Although percutaneous transhepatic gallbladder drainage (PT-GBD) has traditionally served as the first-line non-surgical alternative, its significant morbidity and technical limitations in patients with coagulopathy, massive ascites, or an unsafe percutaneous window have driven the development of endoscopic drainage modalities. This review critically appraises the comparative evidence for two endoscopic gallbladder drainage strategies: endoscopic transpapillary gallbladder drainage (ET-GBD), performed via endoscopic retrograde cholangiopancreatography, and endoscopic ultrasound-guided gallbladder drainage (EUS-GBD), most commonly using lumen-apposing metal stents (LAMS). In appropriately selected high-risk surgical candidates, EUS-GBD with LAMS has emerged as a preferred endoscopic option, supported by high technical success rates, low recurrent AC rates, AGA expert guidance, and FDA regulatory reclassification of the AXIOS stent for gallbladder drainage. Nevertheless, ET-GBD retains distinct clinical indications, including concurrent biliary intervention, large-volume ascites, high bleeding risk, anticipated surgical candidacy, and resource-limited settings. Optimal management requires a systematic, algorithm-driven multidisciplinary approach integrating comorbidity profile, biliary anatomy, cystic duct patency, and institutional expertise.

## 1. Introduction

Acute cholecystitis (AC) is one of the most common gastrointestinal emergencies, and laparoscopic cholecystectomy remains the definitive treatment as recommended by the Tokyo Guidelines 2018 (TG18) [1]. However, a substantial proportion of patients—particularly elderly individuals with major comorbidities, multiorgan dysfunction, or advanced malignancy—are considered poor surgical candidates in whom operative intervention carries prohibitive risk [1,2].

For these high-risk patients, percutaneous transhepatic gallbladder drainage (PT-GBD) has traditionally served as the first-line non-surgical alternative endorsed by TG18; however, it carries significant long-term morbidity, frequent drain-related complications, and high reintervention rates; moreover, PT-GBD may be technically difficult or relatively contraindicated in patients with massive ascites, uncorrected coagulopathy, or an unsafe percutaneous window [2,3]. These limitations have driven the development of two endoscopic drainage modalities: endoscopic transpapillary gallbladder drainage (ET-GBD), performed via endoscopic retrograde cholangiopancreatography (ERCP) with selective cystic duct cannulation; and endoscopic ultrasound-guided gallbladder drainage (EUS-GBD), utilizing lumen-apposing metal stents (LAMS) for transmural cholecystoenterostomy [4,5]. Despite growing evidence supporting both approaches, no definitive consensus exists regarding the optimal endoscopic strategy. This review critically appraises the comparative evidence for ET-GBD and EUS-GBD, examining their technical approaches, outcomes, adverse event profiles, and patient selection principles to guide individualized decision-making in surgically unfit patients with AC.

For clarity, the term EUS-GBD is used throughout this review as an umbrella term encompassing both tubular covered self-expandable metal stent-based EUS-GBD and lumen-apposing metal stent-based EUS-GBD, whereas these two subtypes are specified separately whenever device-specific technical considerations, outcomes, or clinical indications differ.

## 2. Methods: Search Strategy and Evidence Selection

This narrative review focused on endoscopic gallbladder drainage strategies for acute cholecystitis in patients with contraindications to surgery. A literature search was conducted using PubMed/MEDLINE and Scopus, Web of Science and Cochrane Library from database inception through May 2026, with additional manual review of references from relevant guidelines, systematic reviews, meta-analyses, and key original studies. Search terms included combinations of “acute cholecystitis,” “endoscopic transpapillary gallbladder drainage,” “ET-GBD,” “endoscopic ultrasound-guided gallbladder drainage,” “EUS-GBD,” “lumen-apposing metal stent,” “LAMS,” “percutaneous gallbladder drainage,” and “high-risk surgical patients.” Eligible publications included English-language studies addressing ET-GBD, EUS-GBD, LAMS-based gallbladder drainage, comparative outcomes versus PT-GBD, long-term clinical outcomes, procedure-related adverse events, and patient selection strategies. Particular emphasis was placed on recent multicenter studies, comparative and prospective studies, randomized data when available, meta-analyses, and society guidelines most relevant to contemporary clinical decision-making. Selected conference abstracts were also considered when they provided randomized or otherwise clinically relevant comparative data not yet available in full-text publication. As this was a narrative rather than a systematic review, protocol registration, PRISMA-based study selection, formal risk-of-bias assessment, and quantitative meta-analysis were not performed.

## 3. Endoscopic Transpapillary Gallbladder Drainage

### 3.1. Technical Approach of ET-GBD

**Figure 1:**
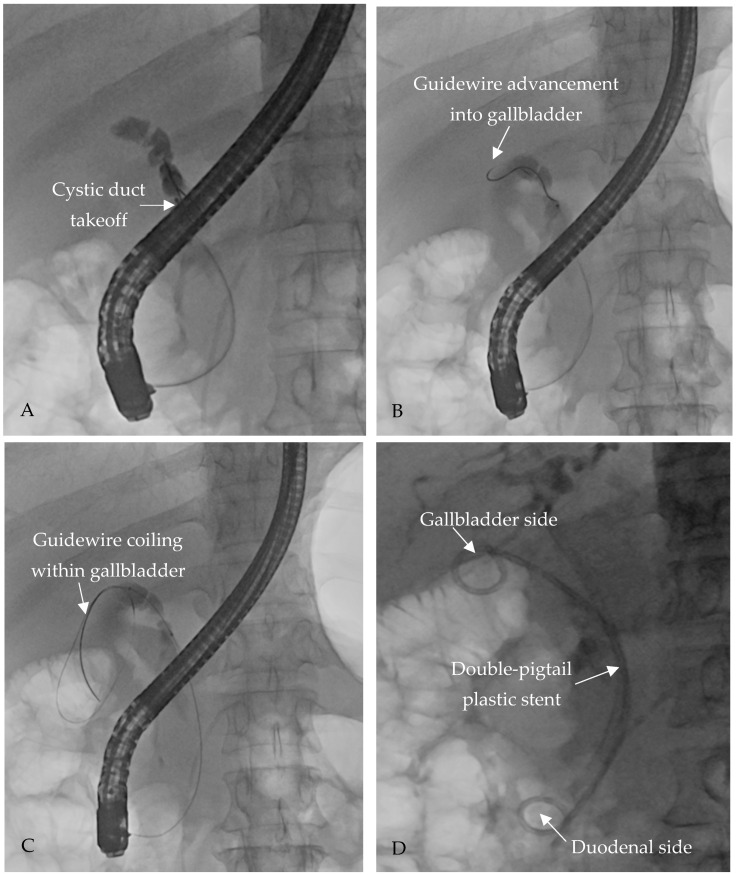
Representative endoscopic transpapillary gallbladder drainage (ET-GBD). (**A**) Cholangiography identifying the cystic duct takeoff. (**B**) Selective guidewire advancement through the cystic duct into the gallbladder. (**C**) Coiling of the guidewire within the gallbladder. (**D**) Placement of a double-pigtail plastic stent from the gallbladder to the duodenum.

ET-GBD was first described in the mid-1980s as an internal drainage approach for surgically unfit patients [6,7]. The procedure utilizes the ERCP platform to decompress the gallbladder through its natural anatomical route: from the duodenal papilla, through the common bile duct (CBD), and selectively into the cystic duct, thereby preserving native biliary anatomy and future surgical candidacy [4,8]. Selective cystic duct cannulation—the critical technical step—requires advancing a 0.025–0.035-inch guidewire through the spiral valves of Heister into the gallbladder lumen under fluoroscopic guidance. Adjunctive techniques, including rotatable sphincterotomes, balloon-occluded cholangiography, and direct cholangioscopy with the SpyGlass DS system (Boston Scientific, Marlborough, MA, USA), can improve technical success in refractory cases. After guidewire access is established, a 5–10 French (Fr) double-pigtail plastic stent (DPPS) is advanced from the gallbladder lumen into the duodenum. Pooled meta-analytic data report technical success rates of 83–88% and clinical success rates of 80–100%, with a post-procedural adverse event rate of approximately 10% [5,9]. Representative procedure steps of the ET-GBD are shown in Figure 1.

### 3.2. Clinical Outcome

Technical failure, occurring in approximately 12–17% of cases, most commonly arises from the inability to selectively cannulate the cystic duct due to acute pericholecystic inflammation, impacted stones at the gallbladder neck, a tortuous or narrow cystic duct, prior biliary instrumentation, or a pre-existing transpapillary biliary metal stent occluding cystic duct access. Additional procedural challenges include difficulty identifying the cystic duct orifice, underlying gallbladder malignancy, and sphincterotomy-related risks in patients requiring anticoagulation [9].

### 3.3. Advantages and Limitations of ET-GBD

ET-GBD offers several clinically important advantages. Because drainage is achieved entirely within the biliary system without transmural fistula creation, gastrointestinal (GI) anatomy remains intact, simplifying subsequent elective cholecystectomy if the patient’s surgical risk improves. A single ERCP session can simultaneously address concurrent CBD stones or biliary obstruction, avoiding an additional procedure. ET-GBD may be favored when transmural puncture is undesirable, such as in patients with significant ascites or high bleeding risk, provided that ERCP is feasible and sphincterotomy can be avoided or safely performed. The procedure requires only ERCP expertise and standard biliary accessories, with substantially lower device cost compared to LAMS, making it broadly accessible in resource-limited settings [4,5,10].

The principal limitation of ET-GBD is its relatively low technical success rate (~83%), substantially inferior to EUS-GBD (~95%) [5,9], primarily due to the inherent difficulty of selective cystic duct cannulation under acute gallbladder inflammation. The procedure carries the well-characterized adverse event (AE) profile of ERCP, including post-ERCP pancreatitis (~2%), endoscopic sphincterotomy (EST)-related bleeding (~1%), and periampullary perforation [9]. ET-GBD is inapplicable when the cystic duct is obstructed by impacted stones, a neoplastic mass, or an indwelling biliary metal stent. The small stent caliber (5–7 Fr DPPS) limits drainage efficacy and precludes stone passage. Most critically, chronic stent-induced cholestasis with duodenal reflux leads to progressive late biliary AEs beyond 2 years, with cumulative late complication rates reaching 27.1% at 3 years in propensity-matched analyses, substantially limiting indefinite ET-GBD as definitive long-term therapy [11,12].

## 4. Tubular Covered Self-Expandable Metal Stent-Based EUS-Guided Gallbladder Drainage

### 4.1. Technical Approach of Tubular cSEMS-Based EUS-GBD

The concept of EUS-GBD was pioneered by Baron and Topazian (2007) and subsequently by Lee et al. and Kwan et al., using double-pigtail plastic stents (DPPS) for transmural cholecystoenterostomy [13,14,15]; Song et al. (2010) further established its feasibility using a single-step DPPS technique [16]. However, the absence of hepatic parenchymal tamponade made bile leakage a persistent concern, and stent patency was limited by the small caliber of DPPS. Covered self-expandable metal stents (cSEMS) were subsequently adopted to address these shortcomings: Jang et al. demonstrated that the superior radial expansion force of cSEMS, applied via both transgastric and transduodenal approaches, more effectively sealed the transmural fistula compared with plastic stents [17]. Despite these advances, standard tubular cSEMS remained prone to axial migration; to mitigate this, co-axial placement of an inner DPPS within the cSEMS was described for bidirectional anchoring, and dedicated anti-migratory cSEMS incorporating flanged ends, such as the BONA-AL stent (Standard Sci-Tech Inc., Seoul, Republic of Korea), were subsequently developed, demonstrating reduced migration rates compared with conventional tubular cSEMS [17,18].

The procedure is performed under monitored anesthesia using a curvilinear array echoendoscope. After identification of an optimal acoustic window free of intervening vessels, confirmed by color Doppler, a 19-gauge fine-needle aspiration (FNA) needle punctures the gallbladder; bile aspiration and fluoroscopic contrast injection confirm intraluminal access. A 0.025–0.035-inch guidewire is coiled within the gallbladder lumen, and the transmural tract is dilated using a bougie catheter or 4 mm balloon before cSEMS deployment under combined EUS/fluoroscopic guidance. Co-axial DPPS placement within the deployed metal stent provides additional bidirectional anchoring to reduce migration risk [17,19].

### 4.2. Advantages and Limitations of Tubular cSEMS-Based EUS-GBD

The principal advantage of tubular cSEMS-based EUS-GBD is independence from cystic duct anatomy, enabling gallbladder decompression when the cystic duct is obstructed by stone impaction, neoplastic occlusion, altered post-surgical anatomy, or an indwelling biliary metal stent. Compared to DPPS, the superior radial expansion force of cSEMS more effectively seals the fresh transmural fistula, reducing bile leakage risk. The wider stent lumen (typically 10 mm) compared to transpapillary plastic stents (5–7 Fr) facilitates more effective drainage of viscous or purulent bile. Long-term retrospective data demonstrate acceptable clinical durability with a median stent patency of approximately 395 days and lower recurrent AC rates compared to ET-GBD [17,20]. The procedure is also applicable when ERCP cannot be performed due to post-surgical anatomy or duodenal obstruction [17,18].

The primary disadvantages relate to stent stability and multi-step procedural complexity. Conventional tubular self-expandable metal stents (SEMS) without anti-migratory features are prone to axial migration—both proximally and distally—which may precipitate bile peritonitis or require emergency reintervention. The sequential steps of FNA puncture, guidewire placement, and tract dilation create multiple opportunities for bile leakage prior to definitive stent sealing. The tubular lumen is generally insufficient for peroral cholecystoscopy or therapeutic stone extraction. Compared to LAMS, tubular cSEMS-based EUS-GBD has a more limited evidence base, lacks specific regulatory approval for this indication, and carries weaker guideline endorsement; current guidelines preferentially recommend LAMS over tubular SEMS when available [21,22].

## 5. LAMS-Based EUS-GBD

### 5.1. Technical Approach of LAMS-Based EUS-GBD

LAMS were originally designed for EUS-guided drainage of peripancreatic fluid collections, including pancreatic pseudocysts and walled-off necrosis (WON) [23]. De la Serna-Higuera et al. (2013) pioneered their application to EUS-GBD, reporting the first dedicated fistula-forming lumen-apposing stent for transmural cholecystoenterostomy [24]; Irani et al. and Walter et al. subsequently confirmed favorable technical and clinical outcomes [25,26]. The fundamental LAMS design—a short saddle-shaped stent body flanked by wide bilateral dumbbell-shaped flanges—provides a robust transmural seal and bidirectional anti-migratory anchoring, eliminating the need for co-axial DPPS. Inner lumen diameters of 10–20 mm enable passage of a standard upper endoscope for peroral cholecystoscopy and stone extraction [27]. A pivotal advance was the electrocautery-enhanced (“Hot”) LAMS delivery system (exemplified by the Hot AXIOS stent; Boston Scientific), enabling single-step simultaneous penetration of the GI wall and gallbladder without preliminary FNA access, guidewire placement, or tract dilation [28]. In August 2023, the U.S. Food and Drug Administration (FDA) formally reclassified the AXIOS LAMS for gallbladder drainage via the de novo 510(k) pathway [22]; the American Gastroenterological Association (AGA) 2023 clinical practice update now recommends LAMS as the preferred stent type for EUS-GBD [21].

**Figure 2:**
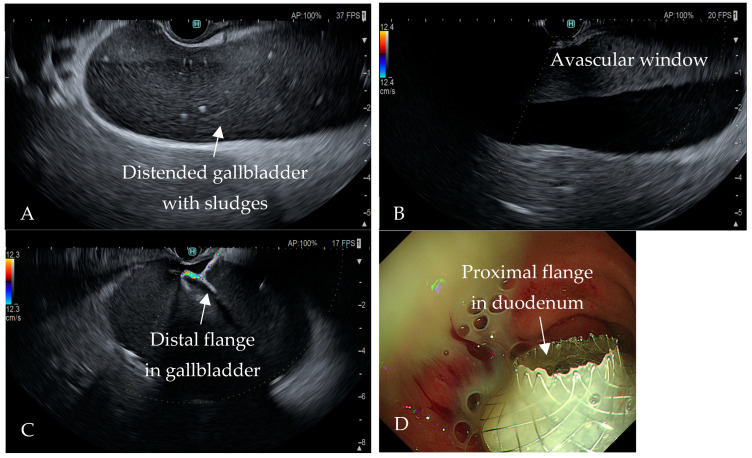
Representative steps of a lumen-apposing metal stent (LAMS)-based endoscopic ultrasound-guided gallbladder drainage (EUS-GBD) (**A**) EUS image showing a distended gallbladder with sludge adjacent to the duodenum. (**B**) Color Doppler confirmation of an avascular access window before cautery-enhanced access. (**C**) Deployment of the distal flange of the LAMS under EUS guidance. (**D**) Endoscopic view showing bile or purulent drainage through the deployed LAMS.

Two principal deployment techniques are employed. The conventional “needle-first” over-the-wire technique involves gallbladder puncture with a 19-gauge FNA needle, guidewire coiling in the gallbladder lumen, balloon tract dilation, and cold LAMS deployment; this approach is preferred for contracted or difficult gallbladders where secure wire access must be maintained throughout. The direct cautery-enhanced Hot-LAMS technique simultaneously penetrates both GI and gallbladder walls in a single step, with immediate distal flange deployment within the gallbladder lumen under EUS guidance, followed by retraction to appose the walls and proximal flange deployment in the GI lumen under direct endoscopic visualization. Optional adjuncts include balloon dilation of the LAMS lumen and co-axial DPPS placement to maintain long-term fistula patency. After fistula maturation at 28–35 days, peroral cholecystoscopy can be performed through the LAMS for direct visualization, stone extraction, and endoscopic laser lithotripsy (ELL) or electrohydraulic lithotripsy (EHL) [21,27]. Representative LAMS-based EUS-GBD are shown in Figure 2.

### 5.2. Advantages and Limitations of LAMS-Based EUS-GBD

LAMS-based EUS-GBD has emerged as an important endoscopic modality for acute cholecystitis in carefully selected high-surgical-risk patients, particularly when appropriate expertise, devices, and patient anatomy are available [21,22]. Its principal procedural advantages derive from the dedicated stent design and transmural drainage route. The bilateral large-diameter flanges provide anti-migratory anchoring and immediate gallbladder-enteric wall apposition, and the Hot-LAMS cautery-enhanced delivery system enables single-step access, thereby reducing procedural complexity, device exchanges, and potential bile leakage during tract creation [28,29]. In pooled data from 18 studies including 701 patients, LAMS-based EUS-GBD achieved technical and clinical success rates of 95.8% and 94.3%, respectively [30]. In addition, the wide inner lumen (10–20 mm) permits peroral cholecystoscopy, stone extraction, and lithotripsy using ELL or EHL, offering a potential route for more definitive gallstone management in patients at high risk for recurrent acute cholecystitis [27]. Meta-analytic data have also shown a lower rate of recurrent acute cholecystitis with EUS-GBD than with ET-GBD (pooled odds ratio 0.33; 95% confidence interval [CI] 0.14–0.79; *p* = 0.01) [5]. Because EUS-GBD does not depend on cystic duct cannulation, it may be particularly useful when transpapillary access is not feasible, provided that a safe EUS window and adequate gallbladder-enteric apposition are present [21,31].

Despite these advantages, the broader applicability of EUS-GBD should be interpreted cautiously. Successful EUS-GBD, particularly when performed using LAMS, requires advanced interventional EUS expertise, which remains unavailable in many institutions worldwide [21,32]. In addition, much of the most favorable evidence has been generated in high-volume tertiary referral centers, and these results may not be directly reproducible in routine clinical practice or in settings with more limited endoscopic infrastructure. Device-related cost and local availability of LAMS may further restrict wider implementation, especially in resource-constrained environments [30]. Procedural outcomes are also influenced by learning-curve effects, as both technical success and procedural safety improve with operator experience [32]. Therefore, the reported advantages of EUS-GBD over alternative drainage strategies should be interpreted in the context of local expertise, available resources, patient anatomy, and institutional practice patterns [21,30,32].

Several procedural and anatomical limitations should also be acknowledged. The transmural approach introduces risks that are absent or less relevant in transpapillary drainage, including perforation, significant gastrointestinal bleeding, bile peritonitis, and self-limiting pneumoperitoneum [30]. LAMS-based EUS-GBD is generally contraindicated in the presence of large-volume ascites, because interposed ascitic fluid prevents secure wall apposition and increases the risk of leakage. Creation of a cholecystoenteric fistula may complicate subsequent cholecystectomy, particularly after duodenal access; therefore, LAMS placement should generally be reserved for patients who are unlikely to undergo surgery unless a multidisciplinary evaluation suggests that future operative management remains feasible [21,33]. Finally, long-term LAMS retention may be associated with stent occlusion from food debris, buried stent syndrome, delayed gastrointestinal bleeding, or migration, requiring individualized post-procedural follow-up and stent-management planning, as discussed in Section 5.3 [30,34]. In settings where advanced EUS expertise, LAMS availability, or safe transmural anatomy is lacking, ET-GBD or PT-GBD may remain the more appropriate drainage strategy.

### 5.3. Post-Procedural LAMS Management: Removal, Exchange, or Permanent Indwelling

The optimal post-procedural management of LAMS after EUS-GBD remains unsettled, with practice varying considerably across institutions. One strategy is planned second-look endoscopy after maturation of the cholecystoenteric fistula, typically at approximately 4–6 weeks, with peroral cholecystoscopy, gallstone clearance when feasible, and subsequent LAMS removal. Expert opinion and observational data have supported this approach—LAMS removal with transluminal endoscopic gallstone clearance—on the basis that long-term metal stent retention may increase the risk of stent-related adverse events (as detailed in Section 5.2) [35]; this strategy also avoids permanent cholecystoenteric fistula formation, potentially simplifying subsequent cholecystectomy if surgical candidacy is preserved [21,33]. After LAMS removal, coaxial placement of a double-pigtail plastic stent to maintain fistula patency while minimizing the risks associated with long-term metal stent retention has been advocated by some experts [35]. A large US multicenter retrospective study of 109 patients reported that LAMS was electively removed in 22% of cases at a median of 38 days post-procedure; the fistula was intentionally left open in 79.2% of removal cases; importantly, LAMS removal was not associated with a significant reduction in recurrent acute cholecystitis (RAC) rates compared with permanent indwelling (4.2% vs. 8.2%; *p* = 0.68), suggesting that routine elective removal does not confer a clear clinical benefit over permanent indwelling [36].

However, routine LAMS removal is not always practical in the population for whom EUS-GBD is most commonly performed. Patients undergoing EUS-GBD are frequently elderly, frail, and permanently unfit for surgery; in such patients, a scheduled repeat endoscopic procedure may be declined or may confer additional procedural and anesthetic risk [35,37]. Moreover, complete gallbladder stone clearance may require more than one cholecystoscopy session and may not be necessary in patients whose primary therapeutic goal is durable internal drainage rather than definitive stone eradication. Multiple prospective and retrospective long-term studies support the safety and efficacy of leaving the LAMS in situ as a destination strategy in carefully selected non-surgical candidates. A single-center retrospective registry of 22 patients with LAMS indwell time ≥12 months reported no LAMS-related adverse events beyond the first year over a median follow-up of 24.4 months, with only 4.5% of patients requiring hospital admission for gallstone-related disease—a rate comparable to readmission rates reported after cholecystectomy in large retrospective series [38]. A large multicenter Italian nationwide study (116 patients; mean follow-up 309 days) confirmed that in the subgroup with follow-up exceeding one year, no recurrence of AC was observed despite none of the patients undergoing LAMS removal, demonstrating durable stent patency and an acceptable long-term safety profile [37]. A prospective multicenter nationwide observational study with 1-year telephone follow-up reported that 54.9% of patients completed the full follow-up period with the LAMS in situ, with an overall 1-year cumulative biliary event risk of 9.7%; pancreatobiliary malignancy was identified as the sole independent risk factor for recurrent biliary events, underscoring the importance of risk stratification in long-term management [35]. A US multicenter retrospective study similarly found that leaving the LAMS in situ did not increase the risk of RAC or delayed adverse events, supporting EUS-GBD as reasonable destination therapy for high surgical-risk patients [36]. A 3-year follow-up registry (50 patients without scheduled removal) demonstrated that although cumulative stent-related adverse events increased over time, all symptomatic complications were confined to the first year; recurrent cholecystitis occurred in only 4% of patients, and no stent-related bleeding or stent-related mortality was observed throughout the entire 3-year observation period [34].

Therefore, LAMS management after EUS-GBD should be individualized rather than standardized. Planned LAMS removal with cholecystoscopic stone clearance, with or without exchange to a double-pigtail plastic stent, may be favored in patients with preserved or potentially recoverable surgical candidacy, younger patients with longer life expectancy, patients with a transgastric access route—in whom symptomatic stent-related adverse events appear substantially more frequent than with the transduodenal approach (66.7% vs. 12.5%; *p* = 0.03) [34]—or those requiring gallbladder clearance for recurrent stone-related events [35,36]. Once a first stent-related adverse event is encountered, endoscopic reassessment with LAMS removal or revision should be strongly considered, given the substantially higher risk of recurrent adverse events in this subgroup [34]. Conversely, permanent LAMS indwelling may be appropriate in permanently inoperable patients, those with limited life expectancy, severe comorbidity, or high procedural risk from repeat endoscopy [34,37,38]. Patients with pancreatobiliary malignancy warrant close biliary surveillance given their elevated risk of recurrent biliary events [35]. Although no standardized surveillance protocol has been established, long-term follow-up should be individualized according to clinical risk, with particular attention to recurrent biliary symptoms, gastrointestinal bleeding, stent occlusion, migration, and buried LAMS syndrome, especially in patients managed with permanent indwelling LAMS [34,35]. Persistent cholecystoenteric fistulas after LAMS removal should also be managed according to the clinical context. In permanently inoperable and asymptomatic patients, observation may be reasonable, whereas symptomatic fistulas, recurrent biliary events, leakage, or planned cholecystectomy should prompt multidisciplinary reassessment and consideration of endoscopic closure, LAMS revision, or exchange to a double-pigtail plastic stent. Because evidence beyond three years remains very limited, very late stent-related events should be regarded as an unresolved evidence gap, and symptom-driven long-term surveillance remains prudent in patients managed with permanent LAMS indwelling [34,35,36,37,38]. In all cases, patients should be monitored for recurrent cholecystitis, cholangitis, pancreatitis, gastrointestinal bleeding, stent occlusion, buried LAMS syndrome, and migration. At present, no randomized trial has definitively established the superiority of routine removal over permanent indwelling; thus, the decision should be guided by surgical candidacy, expected survival, access route, gallstone burden, institutional expertise, and patient preference.

## 6. Current Role of PT-GBD in the Era of Endoscopic Gallbladder Drainage

PT-GBD remains an important option for acute cholecystitis in patients who are poor candidates for urgent cholecystectomy, particularly because it is widely available, technically familiar, and feasible in many hospitals [2,4,8]. Although endoscopic gallbladder drainage, particularly EUS-GBD, may be favored in selected centers with appropriate expertise and devices, PT-GBD continues to play a substantial clinical role because of its broad accessibility, rapid procedural availability, and consistently high technical success [2,3,8,21]. Thus, PT-GBD should no longer be viewed simply as the universal default alternative to surgery, but rather as a pragmatic and still essential option in selected clinical and institutional settings.

Current indications for PT-GBD are most evident when urgent gallbladder decompression is required, and advanced endoscopic drainage is unavailable or impractical. PT-GBD remains particularly relevant in patients with hemodynamic instability, severe sepsis, or major cardiopulmonary comorbidity, because it can often be performed under local anesthesia or minimal sedation. It may also be preferred when institutional workflow allows faster access to interventional radiology than to therapeutic endoscopy, or when a safe percutaneous route is available, coagulopathy can be corrected, and immediate source control is the main priority [2,3,8].

PT-GBD also remains preferable in several practical situations. It is still the most widely accessible gallbladder drainage technique worldwide, including in hospitals without interventional EUS capability. In addition, technical success is generally reported to be approximately 90% to 100% across reviews and comparative studies [4,5,9,31]. Recent meta-analyses and network meta-analyses suggest that PT-GBD and EUS-GBD achieve higher technical success than ET-GBD, largely because ET-GBD is limited by the technical difficulty of selective cystic duct cannulation [9,31]. PT-GBD may therefore remain a reasonable bridging option in critically ill patients when immediate decompression is prioritized over longer-term quality-of-life considerations.

In resource-limited settings, PT-GBD offers clear practical advantages. Unlike EUS-GBD, it does not require dedicated lumen-apposing metal stents, advanced therapeutic EUS platforms, or highly specialized endoscopic expertise. It can often be performed promptly by interventional radiology teams using equipment and skills already available in many hospitals. These factors explain why PT-GBD remains the most realistic and scalable drainage strategy in many parts of the world despite the growing adoption of endoscopic alternatives. Subsequent conversion of PT-GBD to internal EUS-GBD may be considered in selected patients, potentially reducing long-term external tube dependence and improving patient comfort [39].

However, the limitations of PT-GBD are increasingly well recognized. Because it relies on an external catheter, PT-GBD is associated with pain, discomfort, bile leakage, skin problems, catheter dislodgement, and repeated interventions, all of which may substantially reduce quality of life in frail patients [2,3,4]. Recurrent cholecystitis and recurrent biliary events also remain concerns, particularly when PT-GBD is used as definitive therapy rather than as a bridge to cholecystectomy or internal drainage [2,3].

Recent comparative evidence increasingly favors EUS-GBD over PT-GBD when both approaches are available and performed by experienced endoscopists. In the DRAC 1 randomized trial, EUS-GBD achieved similar technical and clinical success but lower 1-year adverse events, fewer reinterventions, fewer unplanned readmissions, less pain, and less recurrent cholecystitis than PT-GBD [40]. A three-way comparative study of LAMS-based EUS-GBD, ET-GBD, and PT-GBD also supports the growing role of endoscopic drainage in selected high-risk surgical patients while underscoring the need to individualize drainage strategy according to local expertise and patient characteristics [41]. Meta-analyses have reached similar conclusions, suggesting that EUS-GBD preserves comparable efficacy while reducing adverse events, reintervention, and readmission, particularly when lumen-apposing metal stents are used [5,9,31].

Overall, the current role of PT-GBD is best understood as selective rather than obsolete. PT-GBD remains highly relevant in resource-constrained settings, in centers without interventional EUS capability, in unstable patients requiring rapid decompression, and when endoscopic access is not feasible. However, where expertise and equipment are available, contemporary evidence suggests that EUS-GBD may offer advantages as a definitive internal drainage modality for suitable high-risk surgical candidates. A balanced modern perspective should therefore recognize PT-GBD as a reliable and widely available drainage strategy whose use should be individualized according to patient stability, anatomy, procedural risk, local expertise, and available resources.

Table 1 is intended to complement the comparative outcomes data in the following section by contrasting the four modalities according to procedural route, practical strengths, limitations, and preferred clinical scenarios.

## 7. Comparative Evidence and Long-Term Outcomes

Available comparative studies evaluating EUS-GBD and ET-GBD are summarized in Table 2. Because completed full-text randomized controlled trials directly comparing EUS-GBD with ET-GBD remain scarce, the table includes the highest currently available comparative evidence, including conference-abstract randomized trials, retrospective comparative studies, and propensity score-matched analyses. To minimize overinterpretation, key methodological features such as study design, sample size, and EUS-GBD stent type are explicitly presented.

Although several comparative studies have reported higher technical success with EUS-GBD than with ET-GBD, these findings should be interpreted cautiously. Most available comparative evidence is based on retrospective analyses, and randomized data remain limited. Moreover, substantial heterogeneity exists across studies with respect to patient selection, disease severity, stent choice, operator expertise, follow-up duration, and endpoint definitions for adverse events and recurrence. Institutional experience and endoscopist proficiency may also have materially influenced procedural and clinical outcomes. Therefore, while EUS-GBD appears promising in comparative studies, the robustness and generalizability of the current evidence remain constrained, and definitive conclusions regarding comparative superiority cannot yet be drawn.

ET-GBD demonstrates favorable short-term biliary event-free rates (99% at 6 months, 92% at 1 year, 76% at ≥2 years), but its durability as definitive long-term therapy is substantially undermined by progressive late biliary AEs [45]. A multicenter propensity score-matched analysis demonstrated comparable short-term AC recurrence rates between ET-GBD and EUS-GBD (3.0% vs. 3.8%; *p* = 1.000) but markedly higher 3-year cumulative late non-cholecystitis AEs with ET-GBD (27.1% vs. 1.4%; *p* = 0.001), driven primarily by bile duct cholangitis secondary to chronic stent-induced cholestasis and duodenal reflux; multivariate analysis confirmed EUS-GBD was independently associated with a significantly longer time to late AE (hazard ratio 0.26; 95% CI 0.10–0.67; *p* = 0.005) [11]. For tubular cSEMS-based EUS-GBD, available retrospective data demonstrate acceptable clinical durability with lower recurrent AC rates compared to ET-GBD; however, the absence of dedicated prospective long-term trials limits the conclusions, and current guidelines preferentially recommend LAMS when available [17,21].

LAMS-based EUS-GBD demonstrates robust long-term clinical durability. A systematic review and meta-analysis by Canakis et al., incorporating 18 studies and 701 patients with a minimum 1-year follow-up, reported a pooled technical success of 95.8%, clinical success of 94.3%, recurrent AC of 4.2%, and reintervention rate of 6.0% [30]. The DRAC 1 randomized controlled trial (RCT) confirmed at 1 year that LAMS-based EUS-GBD achieved significantly lower AE rates than PT-GBD (25.6% vs. 77.5%; *p* < 0.001), with reintervention rates of 2.6% versus 30.0% (*p* = 0.001) [40]. Long-term LAMS retention appears safe in permanent non-surgical candidates: a prospective 3-year registry demonstrated LAMS-related AEs occurring at a median of 674 days post-procedure, with the majority manageable by repeat endoscopy [34]. In patients with preserved operative candidacy, a second-look endoscopy for cholecystoscopic stone clearance at 28–35 days followed by LAMS removal and co-axial DPPS insertion is recommended (as discussed in Section 5.3) [21,46].

## 8. Patient Selection and Practical Decision-Making Algorithm

**Figure 3:**
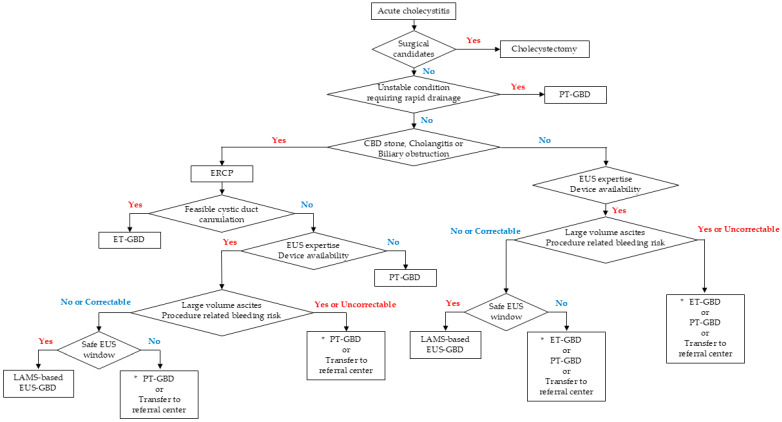
Decision-making algorithm for gallbladder drainage in acute cholecystitis. * Depends on the clinical situation and institutional availability. Abbreviations: ET-GBD, endoscopic transpapillary gallbladder drainage; PT-GBD, percutaneous transhepatic gallbladder drainage; EUS-GBD, endoscopic ultrasound-guided gallbladder drainage; LAMS, lumen-apposing metal stent.

Selection of gallbladder drainage should follow a practical stepwise algorithm that incorporates surgical candidacy, the need for urgent source control, cystic duct patency, ascites, anticoagulation or coagulopathy status, concomitant choledocholithiasis or cholangitis, and local availability of interventional EUS expertise and dedicated devices (Figure 3). Patients who are suitable for surgery should undergo early laparoscopic cholecystectomy, whereas those with prohibitive operative risk or persistent contraindications to anesthesia should be considered for individualized gallbladder drainage after multidisciplinary assessment [4,8,21].

When ERCP is indicated because of concomitant choledocholithiasis, cholangitis, or biliary obstruction, an ERCP-based strategy should generally be considered first. In this setting, ET-GBD is most appropriate when the cystic duct is patent and selective cannulation is feasible. It may also be favored when transmural access is undesirable, such as in patients with large-volume ascites, therapeutic anticoagulation, or high bleeding risk, provided that ERCP can be performed safely and sphincterotomy can be avoided or minimized [4,8,21]. However, ET-GBD is less suitable when the cystic duct is obstructed by an impacted stone, malignant involvement, severe inflammation, or an indwelling transpapillary metal stent.

LAMS-based EUS-GBD may be favored when cystic duct obstruction is suspected or confirmed, when cystic duct patency is uncertain, when ET-GBD has failed, or when durable internal drainage is desired in patients unlikely to undergo subsequent cholecystectomy [21,31,41]. This approach is most suitable when the gallbladder is distended, closely apposed to the gastric antrum or duodenal bulb, and accessible through a safe avascular EUS window, and when appropriate expertise and dedicated devices are available. EUS-GBD should be avoided or deferred in the presence of large-volume ascites, unsafe gallbladder-enteric apposition, uncorrectable coagulopathy, or lack of local expertise.

PT-GBD remains an important alternative when endoscopic drainage is unavailable, impractical, or unsafe. It may be preferred in unstable patients requiring rapid decompression, in centers without interventional EUS capability, in patients in whom endoscopic access is not feasible, or when institutional workflow allows faster drainage through interventional radiology [2,3,4,8]. However, PT-GBD requires a safe percutaneous window and appropriate correction or management of coagulopathy, and it remains limited by external catheter-related morbidity, recurrent interventions, and reduced quality of life when used as definitive therapy [2,3,4,8].

Because patient status may evolve after initial source control, the drainage strategy should be reassessed over time. Interval cholecystectomy should be reconsidered if surgical candidacy improves, particularly after ET-GBD or PT-GBD. Patients treated with EUS-GBD may still proceed to surgery in selected cases, but fistula-related complexity should be anticipated, and surgical teams should be informed before operative planning [33]. Overall, modality selection should be individualized according to patient stability, surgical candidacy, cystic duct anatomy, bleeding risk, ascites, concomitant biliary disease, long-term treatment goals, local expertise, and available resources, as summarized in Figure 3.

## 9. Future Directions

Future development in gallbladder drainage for poor surgical candidates will likely depend on four linked advances: improved stent design, better standardization of technique and training, integration of digital support tools such as artificial intelligence, and stronger prospective comparative evidence. The current literature suggests that technical feasibility is already high in expert hands, but the next stage of progress will require making the procedure safer, more reproducible, and more broadly generalizable across centers.

One major direction is the continued development of next-generation gallbladder stents. Recent device reports show that newer electrocautery-enhanced lumen-apposing metal stent systems are being designed with larger flanges, rounded margins, improved wall apposition, and more controlled delivery mechanisms. The Hot-SPAXUS stent, for example, was developed to simplify deployment and reduce luminal wall trauma, and newer physician-controlled electrocautery-enhanced platforms such as the PLUMBER stent suggest that future devices may offer greater precision and operator control during EUS-guided drainage [47,48]. Future studies should determine whether these design changes can reduce migration, buried-stent events, reintervention rates, and long-term stent dysfunction in patients undergoing EUS-guided gallbladder drainage.

A second priority is procedural optimization and wider dissemination beyond high-volume tertiary centers. Technology reviews on lumen-apposing metal stents emphasize that these procedures remain concentrated in specialized institutions, and that broader adoption will require better patient selection, more refined procedural algorithms, and more explicit strategies for preventing and managing adverse events [49]. Training is also an unresolved issue. Because outcomes of EUS-GBD are influenced by operator experience and institutional resources, future studies should define standardized training pathways, competency benchmarks, and proctoring models [32,49]. Future work should therefore focus on structured credentialing, standardized procedural steps, and multicenter implementation models that can make EUS-guided gallbladder drainage reproducible across different practice settings.

Another emerging area is the integration of artificial intelligence into procedural planning and intraprocedural decision support. Recent reviews in gastrointestinal and biliopancreatic endoscopy suggest that AI may improve endoscopic care through real-time image analysis, anomaly detection, computer-aided diagnosis, workflow optimization, automated reporting, and personalized treatment planning [50,51]. Although direct evidence for AI-assisted EUS-GBD is still limited, these same concepts could be applied to route selection, recognition of difficult anatomy, prediction of technical failure, intraprocedural quality control, and post-procedural risk stratification. At the same time, the current AI literature repeatedly notes important barriers, including data standardization, external validation, explainability, and trustworthiness, all of which must be addressed before routine clinical adoption [50,51].

Robotic-assisted endoscopy may represent another area for future investigation. Direct data on GB drainage are still scarce, but as therapeutic EUS procedures become more technically complex, robotic or computer-assisted platforms may help stabilize endoscope position, improve fine movement control, and reduce operator dependence during transluminal access and stent deployment. Their potential role may be especially relevant in difficult anatomy or in training environments where consistency and precision are critical. At present, this remains a conceptual and translational frontier rather than an evidence-based standard, and dedicated preclinical and clinical studies are needed to clarify whether robotic assistance can meaningfully improve technical success, safety, or training efficiency in EUS-GBD.

Ongoing comparative trials and prospective investigations will be essential to define the next phase of the field. Available reviews repeatedly emphasize the need for larger prospective cohorts, multicenter validation, and comparative analyses rather than continued reliance on retrospective expert-center series [49,50,51]. Future studies should compare newer gallbladder stent platforms against existing lumen-apposing metal stents, clarify the cost-effectiveness of device-specific approaches, and identify which patient subgroups benefit most from EUS-GBD. Important unresolved questions also remain regarding optimal stent dwell time, the role of coaxial plastic stenting in selected patients, standardized follow-up protocols, management of recurrent cholecystitis after initial technical success, and long-term device-specific outcomes. Addressing these issues through prospective investigation would substantially strengthen the translational value of the field.

## 10. Conclusions

Both ET-GBD and EUS-GBD represent effective alternatives to PT-GBD for surgically ineligible patients with acute cholecystitis, but the optimal approach remains context-dependent rather than universally hierarchical. Comparative studies generally suggest higher technical success with EUS-GBD than with ET-GBD, and LAMS-based EUS-GBD currently has the strongest supportive data for durable internal drainage, particularly in experienced centers with appropriate devices and multidisciplinary backup. However, much of the favorable evidence for EUS-GBD has been generated in high-volume expert centers, and these outcomes may not be fully generalized to routine practice, where procedural expertise, device availability, institutional workflow, and cost constraints vary substantially. For this reason, the apparent advantages of EUS-GBD should be interpreted considering expert-center bias and local resource dependence rather than assumed to apply uniformly across all healthcare settings. ET-GBD retains important clinical roles, particularly when concomitant ERCP-based biliary intervention is required, when transmural access is undesirable because of ascites or bleeding risk, when future cholecystectomy remains possible, or when advanced interventional EUS resources are unavailable. Tubular cSEMS-based EUS-GBD remains a reasonable alternative when LAMS is unavailable, but transpapillary drainage is not feasible. Therefore, the most appropriate drainage strategy should be individualized according to patient stability, cystic duct anatomy, surgical candidacy, long-term therapeutic goals, local expertise, and available resources. A systematic, algorithm-driven multidisciplinary approach remains essential to achieving balanced and generalizable clinical decision-making in this complex patient population.

## Figures and Tables

**Table 1:** Practical comparison of gallbladder drainage modalities in poor surgical candidates with acute cholecystitis.

Modality	Typical Route and Device	Main Strengths	Main Limitations	Most Appropriate Situations
ET-GBD	ERCP-guided transpapillary drainage; Usually with a DPPS	Preserves physiologic bile drainage; Simultaneous ERCP-based biliary therapy; Lower device cost; Suitable when future cholecystectomy remains possible	Lower technical success of cystic duct cannulation; ERCP-related adverse events; limited drainage caliber; Less suitable for cystic duct obstruction	Concomitant choledocholithiasis or cholangitis; Preserved cystic duct patency; Anticipated future surgery; Transmural puncture undesirable: ascites, coagulopathy
Tubular cSEMS-based EUS-GBD	EUS-guided transmural drainage using a covered tubular SEMS; Often with coaxial DPPS	Independent of cystic duct patency; Wider lumen than ET-GBD; Effective internal drainage when ERCP access is not possible	Migration risk; Multistep procedure; Weaker evidence base than LAMS; Limited cholecystoscopic access; Less guideline support	Failed or infeasible ET-GBD; Cystic duct obstruction; Altered anatomy; Need for EUS-guided drainage when LAMS is unavailable
LAMS-based EUS-GBD	EUS-guided transmural drainage using lumen-apposing metal stent; Often cautery-enhanced	High technical and clinical success; Strong wall apposition, single-step access possible; Enables cholecystoscopy and stone therapy; Favorable long-term internal drainage data	Requires advanced interventional EUS expertise; Higher device cost; Limited availability; Transmural adverse event risk; Less suitable with large-volume ascites or unsafe apposition	High-risk non-surgical candidates needing durable internal drainage; Failed ET-GBD; Uncertain or obstructed cystic duct; Centers with appropriate EUS expertise and device availability
PT-GBD	Percutaneous transhepatic catheter drainage by interventional radiology	Widely available; High technical success; Feasible in unstable patients; No requirement for advanced endoscopy	External catheter burden; Catheter-related complications (pain, dislodgement, bile leakage, repeat interventions, lower quality of life for long-term use)	Urgent source control; Unstable patients; Lack of interventional EUS capability; Impractical or unsafe endoscopic access, resource-constrained institutions

Abbreviations: ET-GBD, endoscopic transpapillary gallbladder drainage; ERCP, endoscopic retrograde cholangiopancreatography; DPPS, double-pigtail plastic stent; EUS-GBD, endoscopic ultrasound-guided gallbladder drainage; cSEMS, covered self-expandable metal stent; LAMS, lumen-apposing metal stent; PT-GBD, percutaneous transhepatic gallbladder drainage.

**Table 2:** Comparative studies evaluating EUS-guided gallbladder drainage versus endoscopic transpapillary gallbladder drainage in high-risk patients with acute cholecystitis.

Study	Study Design	Patients Analyzed	EUS-GBD Stent	ET-GBD Stent	Technical Success ^‡^	Clinical Success ^‡^	Adverse Events ^‡^	Recurrent Cholecystitis
Oh et al., 2019 [12]	Retrospective; IPTW-adjusted analysis	EUS-GBD 83, ET-GBD 96	anti-migration tubular covered SEMS	Plastic stent	99.3% vs. 86.6%	99.3% vs. 86.0%	7.1% vs. 19.3%	Recurrent AC or cholangitis: 3.2% vs. 12.4% (mean follow-up 21.9 months vs. 20.7 months)
Higa et al., 2019 [10]	Retrospective	EUS-GBD 40, ET-GBD 38	LAMS	Plastic stent	97.5% vs. 84.2%	95.0% vs. 76.3%	17.9% vs. 9.4%	Recurrent AC: 2.6% vs. 18.8% (median follow-up 7 months vs. 5 months)
Nishiguchi et al., 2021 [42]	Retrospective	EUS-GBD 25, ET-GBD 29	FCSEMS with anchoring DPPS	Plastic stent	100% vs. 82.7%	96.0% vs. 79.3%	4.0% vs. 10.3%	Recurrent AC: 0 vs. 4 cases (median follow-up 522 days)
Faknak et al., 2022 * [43]	Randomized trial	EUS-GBD 14, ET-GBD 16	FCSEMS with anchoring DPPS or LAMS	Plastic stent	100% vs. 81.3%	100% vs. 100%	21.4% vs. 12.5%	Recurrent AC: 7.1% vs. 0% (median follow-up 215 days)
Inoue et al., 2023 [11]	Retrospective with PSM analysis	EUS-GBD 90, ET-GBD 90 (matched cohort)	Plastic stent	Plastic stent	96.7% vs. 78.9%	92.0% vs. 94.4%;	7.8% vs. 8.9%;	Recurrent AC: 3.8% vs. 3.0% (mean follow-up 689.8 days vs. 727.6 days)
Chaikajornwat et al., 2024 *^,†^ [44]	Randomized trial	EUS-GBD 26, ET-GBD 29	FCSEMS with anchoring DPPS or LAMS	Plastic stent	100% vs. 82.8%	100% vs. 100%	23.1% vs. 10.3%	Recurrent AC at 1 year: 5.6% vs. 9.1%; Recurrent AC at 1–2 years: 0% vs. 5.0% (median follow-up of 373.5 days)

* Conference abstract only; ^†^ The 2024 Chaikajornwat abstract appears to represent an expanded or updated 2-year outcome analysis from the same research group/trial as the 2022 Faknak abstract; therefore, these data should not be treated as fully independent cohorts; ^‡^ Technical success, clinical success, adverse event values are presented as EUS-GBD versus ET-GBD. Abbreviations: AC, acute cholecystitis; DPPS, double-pigtail stent; ET-GBD, endoscopic transpapillary gallbladder drainage; EUS-GBD, endoscopic ultrasound-guided gallbladder drainage; FCSEMS, fully covered self-expandable metal stent; IPTW, inverse probability of treatment weighting; LAMS, lumen-apposing metal stent; PSM, propensity score matching.

## Data Availability

No new data were created or analyzed in this study.
